# Giant liver abscess with *Streptococcus intermedius* bacteremia treated without any drainage^☆^

**DOI:** 10.1016/j.idcr.2022.e01662

**Published:** 2022-12-20

**Authors:** Yo Ishihara, Sayaka Kaneshiro, Yasukazu Ikehara

**Affiliations:** a Department of General Internal Medicine, Shonan Kamakura General Hospital, Kamakura, Japan; b Department of General Internal Medicine, Uji Tokushukai Hospital, Kyoto, Japan; c Department of Surgery, Ishigakijima Tokushukai Hospital, Okinawa, Japan

**Keywords:** Bacteremia, Liver abscess, Pyogenic, Streptococcus intermedius

## Abstract

A 66-year-old man with hypertension presented with fever which has started three days prior. Computed tomography (CT) revealed the presence of multiple low-density areas in the liver, the largest of which was over 10 cm in diameter, with clear demarcation. *Streptococcus intermedius* was detected in the blood culture, thus we diagnosed suspected liver abscess with bacteremia. Because the patient refused invasive drainage and was not poor general appearance, we had initiated intravenous meropenem followed by ceftriaxone plus metronidazole without any abscess drainage. After 6 weeks antibiotics treatment, liver abscess was almost completely diminished on the CT scan. To the best of our knowledge, this is the first report of a giant liver abscess caused by *Streptococcus intermedius* treated successfully without drainage.

## Introduction

Liver abscess is a rare infectious disease caused by microbes invading the liver parenchyma through blood or bile duct. Though the causative pathogens of liver abscess are different within the regions, in western countries 80 % of liver abscess are bacterial [1]. *Klebsiella pneumoniae*, and *Escherichia coli* are the most common pathogen of liver abscess [2].

Mortality rate of pyogenic liver abscess is high as 15 %, thus adequate diagnosis and treatment are needed [3]. The treatment includes antibiotics and abscess drainage, which is believed to be contribute to reduction of abscess cavity size and decline of mortality rate [2].

In this report, we describe a suspected case of liver abscess caused by *Streptococcus intermedius*, treated with antibiotics alone, without abscess drainage.

## Case presentation

**Fig. 1:**
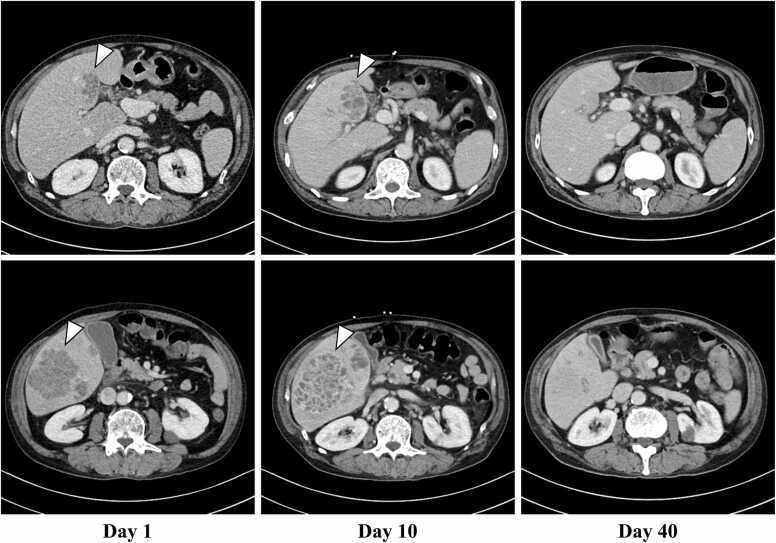
Enhanced Computed tomography scan series of liver abscess. There is multifocal liver abscess (white arrowhead) in S4, S5–6 of the liver on Day 1 and 10. Liver abscess appears to have resolved on day 40.

**Fig. 2:**
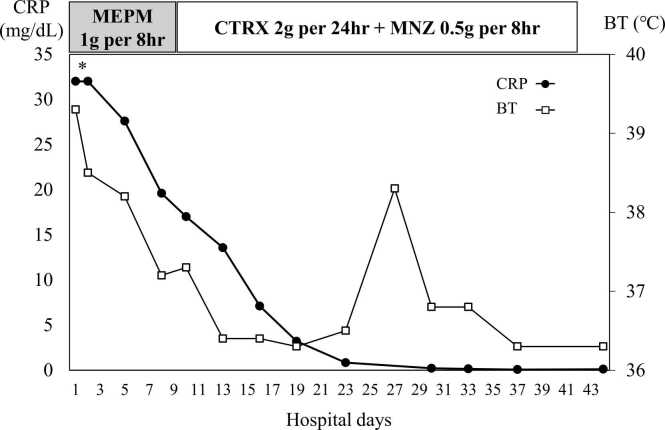
C-reactive protein (CRP) and Body temperature (BT) during treatment. The patient presented with high CRP level on admission day, however it rapidly downtrending after starting antibiotics therapy. BT was transiently elevated by COVID-19 on day 27–29 but remained normal after treatment. (*: Due to the testing equipment, the upper limit for CRP was 32, and the exact value was not known.).

**Fig. 3:**
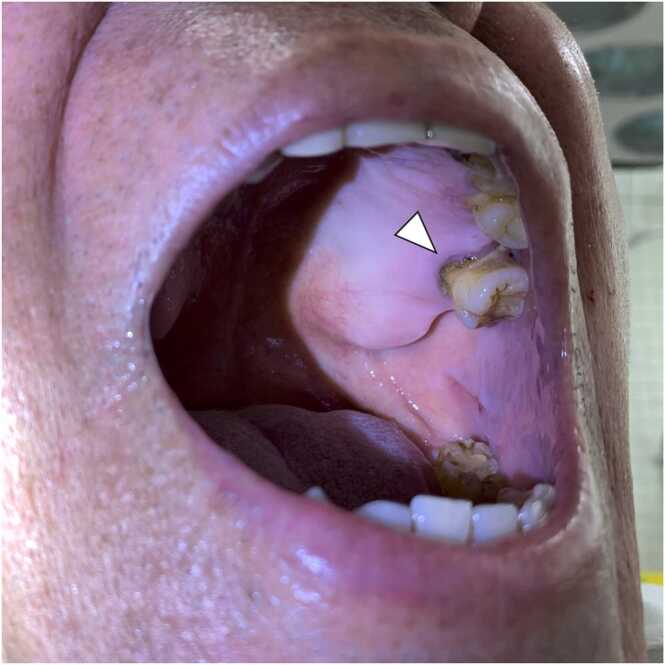
Image of the oral cavity of the patient and the findings of periodontal disease (white arrowheads).

A 66-year-old man visited our hospital complaining of fever with chill which has started three days prior. The patient had a history of appendectomy for appendicitis, hypertension, hyperuricemia, and gastroesophageal reflux disease, which were managed with allopurinol 100 mg once a daily, amlodipine 5 mg once a daily, and vonoprazan 20 mg once a daily. The patient had no history of dental treatment in the past decade. The patient drank about 2 glasses of Awamori, a type of rice wine with 30–40 % alcohol content unique to Okinawa, daily and has smoked 60 cigarettes a day for past 44 years. He denied having sexual intercourse in the past year and denied having homosexual intercourse. Vital signs on arrival showed a temperature of 39.7°, heart rate of 128 beat per minute, blood pressure of 106/62 mmHg, SpO_2_ of 95 % in room air. Physical examination showed that his conscious was clear, and his abdomen was soft and had no significant tenderness. Laboratory tests revealed high hepatic enzymes and C-reactive protein (CRP), and increased white blood cells with neutrocyte dominant (Table 1). Computed tomography (CT) scan revealed that the small hypodense area in S4 and a huge hypodense area with the well-defined boundary of more than 100 mm in diameter in S5–6 (Fig. 1). Initially, we suspected metastatic liver tumor, but we could not rule out liver abscess, thus the patient was admitted to our hospital to start intravenous administration of meropenem 1.0 g per 8 h after collected two sets of blood culture. On the hospital day 8, blood culture on admission day reported *Streptococcus intermedius* was positive in both two sets. In view of the findings of bacteremia and low-density area in the liver on CT scan, we finally diagnosed pyogenic liver abscess. We could not point out any other abscesses through head and body imaging. Based on bacterial susceptibility, antibiotics was changed from meropenem to ceftriaxone 2 g per a day plus metronidazole 500 mg per 8 h. We had recommended percutaneous drainage to the patient, but he refused because of needle phobia, thus we continue antibiotics alone. His fever resolved on the day 5 and CRP was on downtrend with the treatment (Fig. 2). Two sets of blood cultures taken on days 8 and 15 were both negative. On the day 27, the patient again had a fever of 38.3 degrees, and tested for SARS-CoV-2 antigen was positive. Because of the outbreak in the hospital, he was diagnosed with nosocomial COVID-19, however his symptom was recovered within 2 days. On day 29, blood test showed white blood cell count of 2900/µL, whose percentages of neutrophil was 40.7 %. Because of the gradual decrease in white blood cell count, drug-induced neutropenia was suspected, and vonoprazan was discontinued and filgrastim 75 µg was administered. On the day 40, neutrophil counts were recovered to 4400/µL with 52 % of neutrocyte. A total of 6 weeks of antimicrobial therapy was completed on day 40, and we confirmed that liver abscess had been almost completely diminished on enhanced CT scan (Fig. 1). Esophagogastroduodenoscopy (EGD) and colonoscopy (CS) were performed to search the bacterial entry. There was no suspicious findings of on esophagus and stomach, but there were the brownish stains under the patient’s teeth, suggesting that periodontal disease (Fig. 3). CS revealed diverticulum and 0-Isp polyp on sigmoid colon; pathological diagnosis was low grade tubular adenoma. After discharge from our hospital on day 42, the patient was followed up in an outpatient clinic for 3 months. There was no recurrence of liver abscess and no elevation of CRP.

**Table 1:** Laboratory data on the day of admission.

Hematology			Chemistry					
White blood cells	14,500	/µL	Total bilirubin	2.3	mg/dL	Hemoglobin A1c	6.0	%
Lymphocyte	3.2	%	Direct bilirubin	1.0	mg/dL	Total Protein	6.6	g/dL
Neutrocyte	87.7	%	Amylase	54	U/L	Albumin	2.6	g/dL
Eosinocyte	0	%	Aspartate aminotransferase	134	U/L	Sodium	125	mmol/L
Basocyte	0.1	%	Alanine aminotransferase	91	U/L	Potassium	3.1	mmol/L
Red blood cells	446	× 10^4^/µL	Lactate dehydrogenase	323	U/L	Chloride	88	mmol/L
Hemoglobin	14.6	g/dL	γ-Glutamyl transpeptidase	202	IU/L	Calcium	8.3	mg/dL
Hematocrit	36.9	%	Alkaline phosphatase	104	IU/L	C-reactive protein	> 32.0	mg/dL
Mean corpuscular volume	94.6	fL	Blood urea nitrogen	22.5	mg/dL	Procalcitonin	> 10.0	ng/mL
Platelets	14.5	× 10^4^/µL	Serum creatinine	1.42	mg/dL			
			Estimated glomerular filtration rate	40.0	mL/min/1.73 m^2^

## Discussion

We described the case of a patient with giant liver abscess treated with intravenous antibiotics without drainage. On initial CT scan, metastatic liver tumor was suspected as the differential diagnosis of liver abscess. Blood culture revealed *Streptococcus intermedius* bacteremia, thus we had diagnosed suspected pyogenic liver abscess and accomplish successful treatment.

Treatment for liver abscess includes antibiotics and drainage. Antibiotics should be started promptly followed by percutaneous abscess drainage, which enable to identify the causative pathogens, and evacuation of the contains of abscess. Abscess drainage is performed in most case because it is believed to contribute to decreased mortality rate. For successful treatment of liver abscess, sonographic or CT guided needle puncture with aspiration drainage is the first line therapy [1]. There are two types of percutaneous drainage: percutaneous catheter drainage and percutaneous needle drainage. Both of the methods are safe, and percutaneous catheter drainage is more effective because of higher success rate, and reduction of the time for 50 % reduction in abscess cavity size [4]. Surgical intervention is needed in cases of drainage failure, abscess rupture, and failure of antibiotics treatment [2]. In the past, antibiotics were administered without drainage, but poor prognosis were reported [5]. If the liver abscess is huge, more than 5 cm in size, antibiotic treatment alone increases the risk of serious complications [1]. However, studies include cases of a 10 cm giant pyogenic liver abscess treated with antimicrobials alone has been reported in the past [6], [7]. In this case, we could not perform drainage because of a patient refusal. Since the patient had no histories of diabetes, alcoholism, and malignancy, thus we decided to treat him with antibiotics alone, as we did not think he had a high-risk disease. The abscess wall showed a contrast effect, and the abundant blood flow may have contributed to the success of the treatment. Under limited situations, with caution for severity, treatment with antibiotics only, without drainage, appeared to be an option. To the best of our knowledge, this is the first case report of a giant liver abscess caused by *Streptococcus intermedius* treated with antibiotics alone.

*Streptococcus intermedius*, one of the species in *Streptococcus anginosus* group, is causative pathogens of pyogenic liver abscess [8]. *Streptococcus intermedius* is found in normal flora in respiratory, gastrointestinal, and genitourinary tracts, and thought to be cause of abscess in liver, brain, meninges, heart, sinuses, lungs, spleen [9]. In a cohort study, 2.2 % of liver abscess were caused by *Streptococcus intermedius* [10]. Dental cleanings, colon cancer, gastric cancer, acupuncture and moxibustion treatment, acute cholangitis, are reported to be entry site of the bacteria [9], [11], [12], [13], [14], [15]. Dental manipulation, sinusitis, diabetes, heavy alcohol consumption, congenital heart disease and heart-related conditions, and cancer are the risk factors of *Streptococcus intermedius* infections [8]. Daily alcohol consumption was thought to be a risk factor for periodontal disease [16]. In this case, the patient had history of daily alcohol consumption dental problems and periodontal disease as a risk factor. In this case, the patient has a history of dental disease due to daily alcohol consumption and periodontal disease as a risk factor. Since other differential diagnoses are negative, it is assumed that periodontal disease is the entry point for the bacteria.

## Conclusion

Here we present a limited case of a liver abscess caused by *Streptococcus intermedius* that was treated with antibiotics alone without drainage. Physicians consider the possibility of success only antibiotics therapy to giant liver abscess without drainage, in low-risk patients with difficulty in drainage treatment for variable reason.

## CRediT authorship contribution statement

**Yo Ishihara:** Conceptualization, Writing – original draft, Writing – review & editing, Visualization, Project administration. **Sayaka Kaneshiro**: Writing – review & editing. **Yasukazu Ikehara**: Writing – review & editing.

## Sources of funding

None.

## Ethical approval

N/A.

## Consent

Written informed consent was obtained from the patient for publication of this case report and accompanying images. A copy of the written consent is available for review by the Editor-in-Chief of this journal on request.

## Conflicts of interest

The authors declare that they have no conflicts of interest.
